# Pentacyclic triterpenoid ursolic acid interferes with mast cell activation *via* a lipid-centric mechanism affecting FcεRI signalosome functions

**DOI:** 10.1016/j.jbc.2022.102497

**Published:** 2022-09-15

**Authors:** Gouse M. Shaik, Lubica Draberova, Sara Cernohouzova, Magda Tumova, Viktor Bugajev, Petr Draber

**Affiliations:** 1 Department of Signal Transduction, Institute of Molecular Genetics of the Czech Academy of Sciences, Prague, Czech Republic; 2 Department of Biochemistry, College of Science, King Saud University, Riyadh, Saudi Arabia

**Keywords:** mast cell, immunoglobulin E, plasma membrane, lipid raft, signal transduction, tumor necrosis factor, tyrosine kinase, AF, Alexa fluor, BMMC, bone marrow-derived mast cells, BSA, bovine serum albumin, BSS, buffered salt solution, DMSO, dimethyl sulfoxide, DRM, detergent-resistant membrane, FRAP, fluorescence recovery after the photobleaching, geo MFI, geometric mean of fluorescence intensity, HRP, horseradish peroxidase, MβCD, methyl-β-cyclodextrin, PG, prostaglandin, PI, propidium iodide, qPCR, quantitative PCR, RBL, rat basophilic leukemia, SCF, stem cell factor, TNP, 2,4,6-trinitrophenol, UA, ursolic acid

## Abstract

Pentacyclic triterpenoids, including ursolic acid (UA), are bioactive compounds with multiple biological activities involving anti-inflammatory effects. However, the mode of their action on mast cells, key players in the early stages of allergic inflammation, and underlying molecular mechanisms remain enigmatic. To better understand the effect of UA on mast cell signaling, here we examined the consequences of short-term treatment of mouse bone marrow-derived mast cells with UA. Using IgE-sensitized and antigen- or thapsigargin-activated cells, we found that 15 min exposure to UA inhibited high affinity IgE receptor (FcεRI)–mediated degranulation, calcium response, and extracellular calcium uptake. We also found that UA inhibited migration of mouse bone marrow-derived mast cells toward antigen but not toward prostaglandin E_2_ and stem cell factor. Compared to control antigen-activated cells, UA enhanced the production of tumor necrosis factor-α at the mRNA and protein levels. However, secretion of this cytokine was inhibited. Further analysis showed that UA enhanced tyrosine phosphorylation of the SYK kinase and several other proteins involved in the early stages of FcεRI signaling, even in the absence of antigen activation, but inhibited or reduced their further phosphorylation at later stages. In addition, we show that UA induced changes in the properties of detergent-resistant plasma membrane microdomains and reduced antibody-mediated clustering of the FcεRI and glycosylphosphatidylinositol-anchored protein Thy-1. Finally, UA inhibited mobility of the FcεRI and cholesterol. These combined data suggest that UA exerts its effects, at least in part, *via* lipid-centric plasma membrane perturbations, hence affecting the functions of the FcεRI signalosome.

Antigen-triggered activation of mast cells followed by the release of various inflammatory mediators, such as histamine, chemokines, cytokines, and products of arachidonic acid metabolism, are important events initiating allergic inflammation. In this process, the high affinity IgE receptor (FcεRI) complex localized at the plasma membrane plays a key role. FcεRI is a tetramer composed of the α subunit, which binds IgE, two γ subunits, which generate the downstream signal through the process initiated by tyrosine phosphorylation, and one β subunit, which amplifies the activation signals. FcεRIs, various enzymes, ion channels, and transmembrane adapter proteins involved in the FcεRI signaling form a functional complex embedded within the phospholipid bilayer of the plasma membrane, called the FcεRI signalosome. The FcεRI signalosome is a highly dynamic complex that interacts with and is regulated by various plasma membrane and cytoplasmic components and plays a crucial role in propagating the signal from FcεRI into the cytoplasm and the nucleus. This leads to degranulation of preformed secretory granules and *de novo* production and release of cytokines and other inflammatory mediators (1, 2, 3, 4, 5).

Recognition of the critical role of mast cells in allergic inflammation was the driving force in the search for new compounds that prevent mast cell activation, degranulation, and production of inflammatory mediators (3, 6). One natural compound with anti-inflammatory properties under investigation is ursolic acid (UA; 3β-hydroxy-12-ursen-28-ic acid; Fig. 1*A*), a pentacyclic triterpene that is mainly found but not limited to peels of fruits and some herbs (7, 8, 9). Several beneficial effects of UA and its derivatives have been demonstrated in the treatment of various diseases, including cancer (10, 11), obesity (12), glucose metabolism, and diabetes (13, 14). UA is also known for its hepatoprotective (15, 16), neuroprotective, and antidepressant (17, 18) effects. The anti-inflammatory activity of UA was examined in edema (8, 19), passive cutaneous, and systematic anaphylaxis (20) and has been mainly attributed to its ability to affect extracellular signal-regulated kinase (ERK), NF-κB signaling pathways (21, 22), and the activity of protein tyrosine phosphatase (PTP)1B (23). However, there are no data on the molecular mechanisms of the inhibitory effect of UA on the earliest stages of FcεRI-signalosome activation.

**Figure 1:**
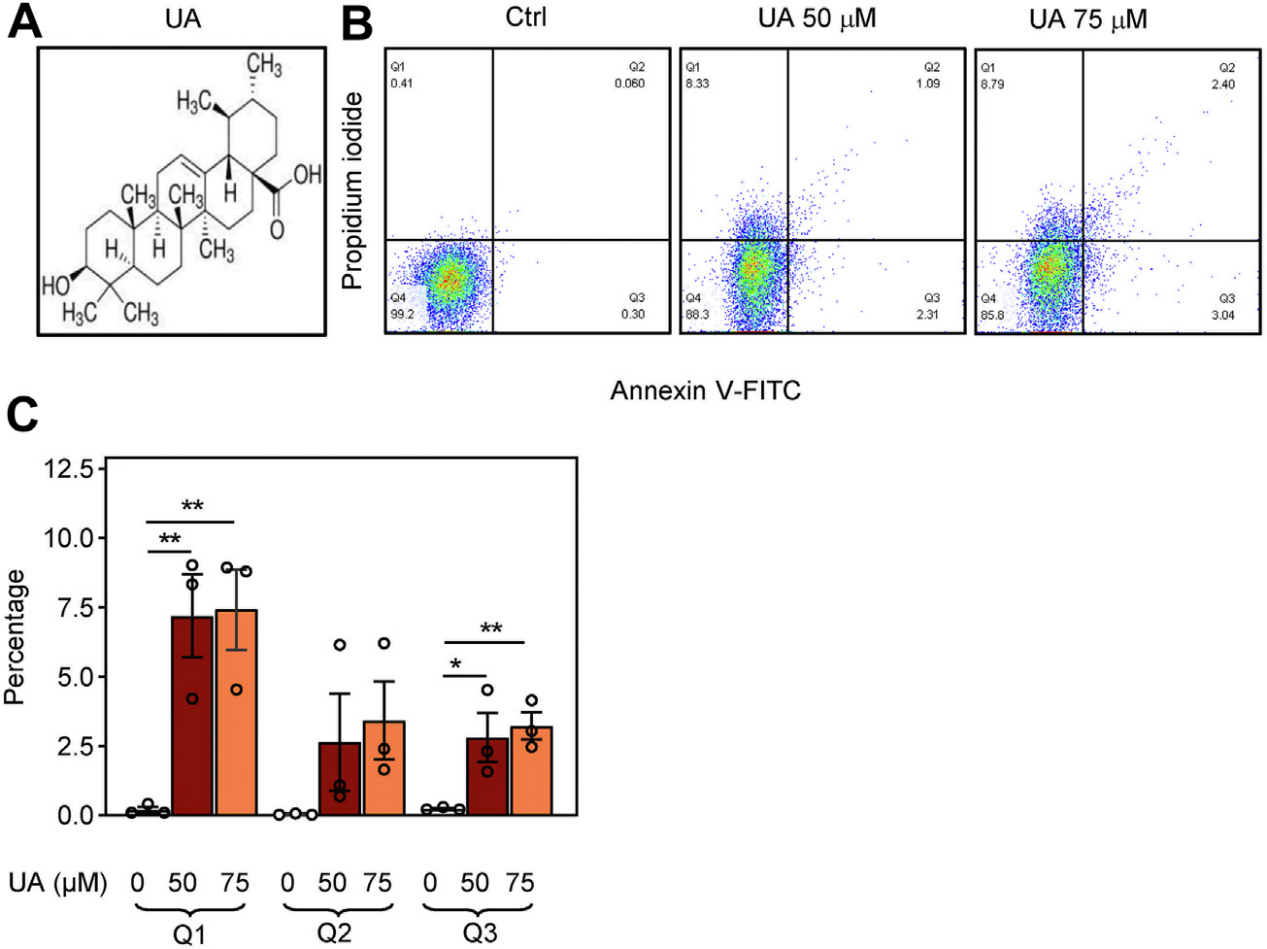
**Increased phosphatidylserine plasma membrane expression and propidium iodide (PI) staining in BMMCs exposed to UA.***A*, chemical structure of UA. *B*, flow cytometry analysis of cells exposed for 15 min to BSS-0.1% BSA supplemented with 0.1% DMSO, used as a control (Ctrl), or UA at a concentration of 50 μM or 75 μM. The cells were stained for phosphatidylserine expression with annexin V-FITC conjugate and for membrane integrity with propidium iodide. The percentage of positive cells in individual quadrants (Q) from a typical experiment is also shown. *C*, data from three independent experiments performed as in (*B*) were analyzed, and mean values ± SEMs in quadrants Q1, Q2, and Q3 were calculated. Statistical significance of the intergroup differences is also shown. BMMC, bone marrow-derived mast cells; DMSO, dimethyl sulfoxide; UA, ursolic acid.

There are two fundamental theories of UA action on cells, protein-centric and lipid-centric. The protein-centric theory proposes that UA interacts with specific proteins and, in this way, affects their properties. Several UA-binding proteins have been described, including PTB1B (23), BCL2 (24), and α-glucosidase (25). The lipid-centric theory postulates that UA, similarly to, for example, anesthetics (26), dissolves in and interacts with cellular lipids and acts by changing the physical properties and spatial arrangement of plasma membrane components. This theory is supported by X-ray diffraction, differential scanning calorimetry, ^31^P NMR, Raman spectroscopy, and fluorescence anisotropy using liposomes of defined lipid composition (27, 28, 29, 30, 31).

In this study, we used mouse bone marrow-derived mast cells (BMMCs) and rat basophilic leukemia (RBL) cells and examined the sensitivity of FcεRI-initiated signaling events in the presence or absence of UA. We focused on tyrosine phosphorylation of the FcεRI-β subunit and other kinase and phosphatase substrates, calcium response, degranulation, and production and secretion of tumor necrosis factor (TNF-α). We also examined UA-induced changes in chemotaxis toward various chemoattractants and changes in the physical properties of the plasma membrane. Our data indicate that UA interferes with the earliest signaling events in FcεRI triggering. This effect seems to be, at least in part, the result of UA-induced changes in the properties of the plasma membrane. In this way, our data support the lipid-centric hypothesis of UA action at the earliest stages of mast cell signaling.

## Results

### Short-term exposure to UA inhibits Fc**ε**RI-induced degranulation and calcium response

To examine the effects of short-term UA on antigen-induced activation of mast cells, we used BMMCs from C57BL/6 mice cultured for 8 to 12 weeks in culture media supplemented with stem cell factor (SCF) and interleukin (IL)-3. More than 95% of the cells were mast cells, as deduced from the expression of both FcεRI and KIT receptors (not shown). Exposure of the BMMCs for 15 min to UA at a concentration up to 75 μM had no toxic effect, as determined by the trypan blue dye exclusion test (not shown). Detailed analysis by flow cytometry (Fig. 1, *B* and *C*) showed that a small fraction of BMMCs exhibited significantly increased propidium iodide (PI) fluorescence (Q1) 15 min after exposure to 50 μM UA (7.2% ± 1.2%; mean ± SEM) or 75 μM UA (7.4% ± 1.1%). A small fraction of BMMCs also showed increased cell surface expression of phosphatidylserine, as reflected by an increased number of annexin V-FITC positive cells (Q3) 15 min after exposure to 50 μM UA (2.6% ± 0.4%) or 75 μM UA (3.1% ± 0.3%). Interestingly, only a small number of cells simultaneously exhibited both markers (Q2) after the treatment with 50 μM UA (2.5% ± 1.0%) or 75 μM UA (3.2% ± 0.8%). These and other data (see later) suggested that short-term pretreatment of the cells with UA at the concentrations used had minimal toxic effects. The observed increased staining with PI and annexin V-FITC could reflect changes in the plasma membrane caused by an interaction of UA with the plasma membrane lipids and not because of a toxic effect of the drug (32, 33).

**Figure 2:**
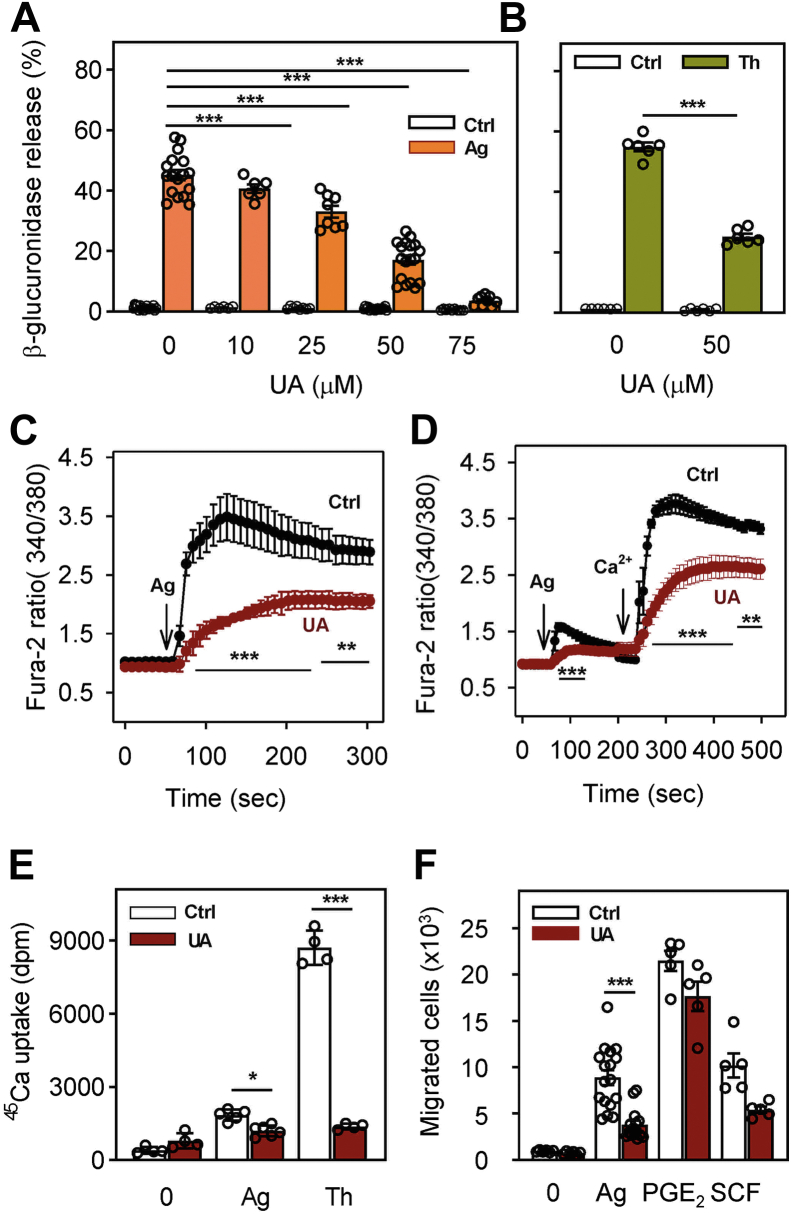
**UA inhibits antigen-induced mast cell degranulation, calcium responses, and chemotaxis.***A*, IgE-sensitized BMMCs were preincubated for 15 min with vehicle (0.1% DMSO) or various concentrations of UA (10–75 μM), which was also present during antigen-mediated activation. Fifteen minutes after adding antigen (Ag; 250 ng/ml TNP-BSA in BSS-0.1% BSA) or BSS-0.1% BSA alone (Ctrl), degranulation was quantified by analysis of β-glucuronidase levels. *B*, BMMCs were exposed for 15 min to 50 μM UA or vehicle (0.1% DMSO). The cells were activated with 1 μM thapsigargin (Th) or were exposed to BSS-0.1% BSA (Ctrl). Degranulation was determined after 15 min as above. *C*, IgE-sensitized and Fura-2-loaded BMMCs were preincubated with 50 μM UA or vehicle (0.1% DMSO; Ctrl) in BSS containing 1.8 mM Ca^2+^. After 15 min, the cells were activated by antigen (Ag; 250 ng/ml TNP-BSA) at the time point indicated by an *arrow*. [Ca^2+^]_i_ was monitored for the indicated time intervals as changes in fluorescence ratios of 340/380 nm. *D*, the cells were treated the same way as in (*C*), except that BSS without calcium was used, and 1.8 mM calcium was added at the indicated time interval after antigen triggering (arrow, Ca^2+^). *E*, IgE-sensitized cells were treated for 15 min with vehicle (Ctrl) or 50 μM UA and then activated with antigen (250 ng/ml TNP-BSA) or thapsigargin (1 μM) in the presence of extracellular ^45^Ca^2+^ (1 mM). After 15 min at 37 °C, the reaction was terminated by centrifugation of the cells through 12% BSA in PBS and cell-bound radioactivity was determined. *F*, the effect of UA on BMMC chemotaxis was determined in transwell chambers. IgE-sensitized cells were treated for 15 min with vehicle (Ctrl) or 50 μM UA and then transferred into *upper* wells of transfer chambers. Migration of the cells toward antigen (250 ng/ml), PGE_2_ (100 nM), or SCF (100 ng/ml), present together with 50 μM UA in bottom wells, was determined after 6 h. Means ± SEMs were calculated from 3 (*C* and *D*) to 4 to 17 (*A*, *B*, *E*, and *F*) independent experiments. Statistical significance of intergroup differences is also shown. BMMC, bone marrow-derived mast cells; BSA, bovine serum albumin; BSS, buffered salt solution; DMSO, dimethyl sulfoxide; TNP, 2,4,6-trinitrophenol; UA, ursolic acid.

Pretreatment of IgE-sensitized BMMCs for 15 min with UA at 25 to 75 μM significantly inhibited antigen-induced degranulation in a dose-dependent way (Fig. 2*A*). Almost complete inhibition of degranulation was observed with 75 μM UA. It should be noted that at all concentrations tested, UA did not affect the spontaneous release of β-glucuronidase from nonactivated cells. This observation supports the notion that short-term exposure of BMMC to UA at concentrations up to 75 μM is not toxic to BMMCs. In further experiments, we used UA at a concentration of 50 μM, which inhibited degranulation by approximately 50%.

Mast cells could also be activated by thapsigargin, which induces the release of Ca^2+^ from intracellular stores by inhibiting the endoplasmic reticulum Ca^2+^ ATPase and, in this way, bypasses all FcεRI-mediated early activation steps. When BMMCs were exposed to 1 μM thapsigargin, strong degranulation was observed (Fig. 2*B*). Pretreatment with 50 μM UA for 15 min significantly inhibited this degranulation (Fig. 2*B*).

To determine whether the calcium response is also affected by 15 min exposure to UA, we monitored concentrations of free cytoplasmic calcium [Ca^2+^]_i_ with a Fura-2 probe. We found that 50 μM UA significantly inhibited the antigen-induced calcium response in cells kept in the presence of 1.8 mM calcium (Fig. 2*C*). Cells activated by antigen in the absence of extracellular calcium exhibited only a small increase in [Ca^2+^]_i_, which was significantly inhibited by UA. After the addition of 1 mM calcium, a rapid rise in [Ca^2+^]_i_ was observed in control cells, as described in previous studies (34, 35) but not in UA-treated cells (Fig. 2*D*). The reduced calcium response in UA-treated cells was, at least in part, caused by reduced uptake of extracellular Ca^2+^, as determined by ^45^Ca-binding assay. In this assay, antigen-induced activation led to an increase in ^45^Ca uptake, which was significantly inhibited by 50 μM UA (Fig. 2*E*). When compared to antigen-activated cells, thapsigargin-activated cells exhibited higher ^45^Ca uptake, which was inhibited by 50 μM UA to the levels comparable to those in UA-treated and antigen-activated cells (Fig. 2*E*).

### Short-term exposure to UA inhibits chemotaxis toward antigen but not towards PGE_2_ and SCF

An essential aspect of mast cell physiology is chemotaxis toward various chemoattractants (36). Next, we, therefore, compared chemotaxis of vehicle- and UA-treated cells toward antigen, prostaglandin (PG)E_2_, and SCF. In the transwell migration assay, 15 min pretreatment of IgE-sensitized BMMCs with 50 μM UA resulted in significantly reduced chemotaxis toward antigen (Fig. 2*F*). When PGE_2_ was used as a chemoattractant, chemotaxis was higher but was not significantly inhibited by 50 μM UA. No significant inhibition by UA was also observed when SCF was used as a chemoattractant (Fig. 2*F*). These data indicate that UA selectively blocks chemotaxis toward antigen and not the general ability of the cells to migrate toward chemoattractants.

### Short-term exposure to UA promotes the production of TNF-α but inhibits its release from antigen-activated cells

**Figure 3:**
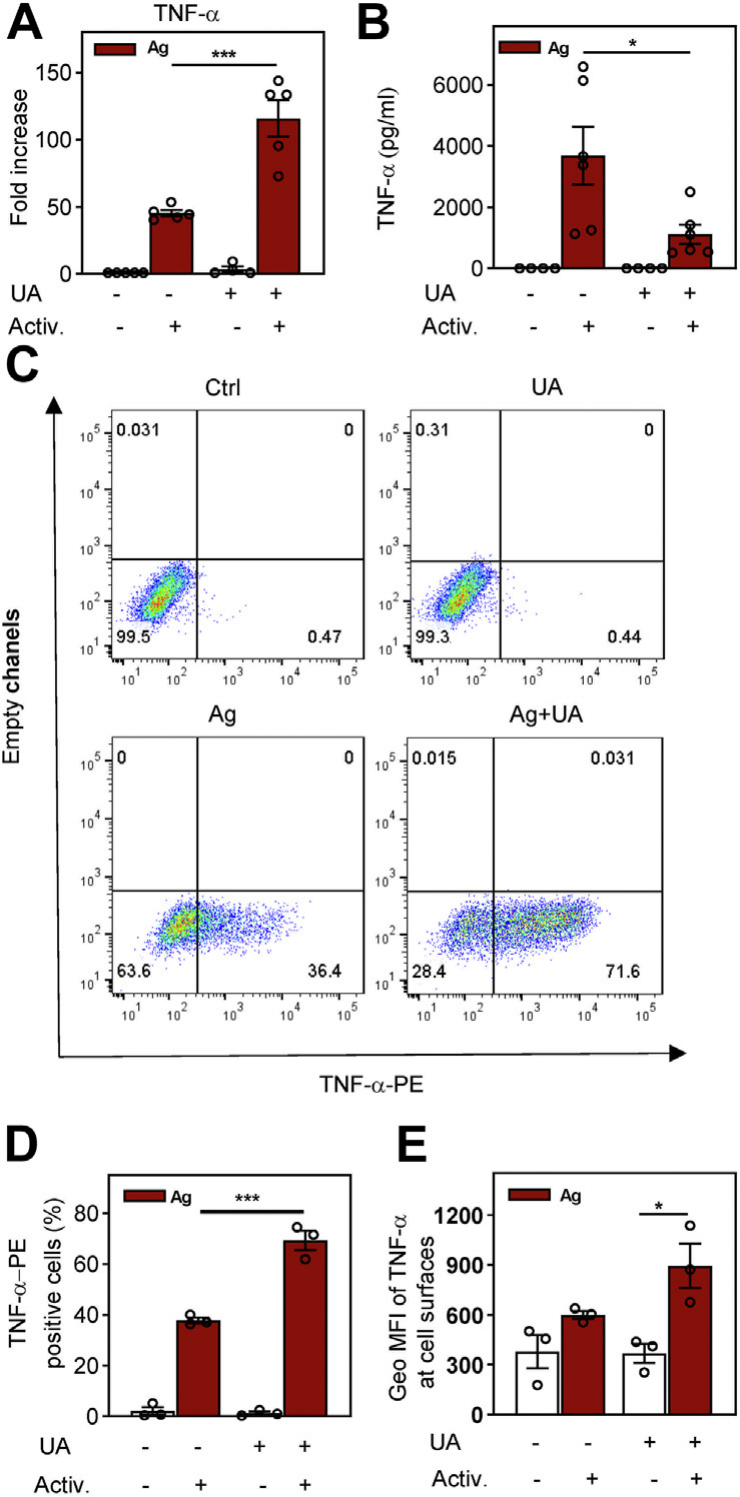
**Enhanced production but reduced cell release of cytokine TNF-α in UA-treated and antigen-activated cells.***A*, IgE-sensitized cells of the BMMC cell line were preincubated for 15 min with vehicle (0.1% DMSO; UA -) or 50 μM UA (+) and then activated (+) or not (−) with antigen (TNP-BSA; 250 ng/ml). After 1 h, RNA was isolated, and mRNA for TNF-α was quantified by RASL-qPCR. Data are presented as fold changes in the TNF-α mRNAs normalized to the expression levels of GAPDH mRNA. *B*, the levels of TNF-α released into the supernatant from the cells treated as in (*A*) for 4 h and quantified by bead-based immunoassay. *C*, flow cytometry analysis of the total cellular TNF-α in nonactivated or antigen-activated cells in the presence or absence of 50 μM UA. *D*, statistical evaluation of TNF-α positive cells (from the PE-positive quadrants as in *C*). Means ± SEMs were calculated from three to six independent experiments. Statistical significance of intergroup differences is also shown. BMMC, bone marrow-derived mast cells; BSA, bovine serum albumin; DMSO, dimethyl sulfoxide; TNP, 2,4,6-trinitrophenol; UA, ursolic acid.

Antigen-mediated activation of BMMCs leads to enhanced secretion of cytokine gene products (1, 2). To determine whether UA has any effect on this process, we analyzed the levels of TNF-α mRNA in IgE-sensitized BMMCs treated with 50 μM UA for 15 min and then activated with antigen in the presence of UA for 60 min. We found that treatment with UA in nonactivated cells did not affect basal levels of TNF-α mRNA (Fig. 3*A*). Surprisingly, in antigen-activated cells, the amount of TNF-α mRNA in UA-treated cells was significantly higher compared to the cells treated with vehicle (0.1% dimethyl sulfoxide [DMSO]).

In UA-treated and antigen-activated cells, the amount of TNF-α released to the supernatant was significantly reduced when compared to antigen-activated control cells (Fig. 3*B*). To determine whether the reduced amount of secreted TNF-α in UA-treated cells reflects the decreased amount of total cellular TNF-α, we examined the presence of TNF-α by flow cytometry in saponin-permeabilized cells. We found a significantly increased percentage of TNF-α–positive cells in the UA-treated and antigen-activated cells than in activated control cells (Fig. 3, *C* and *D*). In antigen-activated and vehicle-treated cells, the geometric mean of fluorescence intensity (geo MFI) was 1143 ± 70 (mean ± SEM). In cells treated with UA and activated with antigen, the geo MFI was increased 3.7 times to 4250 ± 506; the difference was significant (*p* < 0.002). When the assay was performed with cells not permeabilized with saponin to measure surface TNF-α, we found only 1.4 higher geo MFI of TNF-α at the cell surfaces of activated and UA-treated cells when compared to activated control cells, and the difference was not significant (Fig. 3*E*). The data suggested that the reduced secretion of TNF-α is not caused by an accumulation of pro-TNF-α at the plasma membrane due to reduced activity of ADAM metallopeptidase, an enzyme responsible for pro-TNF-α cleavage and mature TNF release (37).

### Different effects of UA on tyrosine phosphorylation of various signal transduction proteins in Fc**ε**RI-activated cells

**Figure 4:**
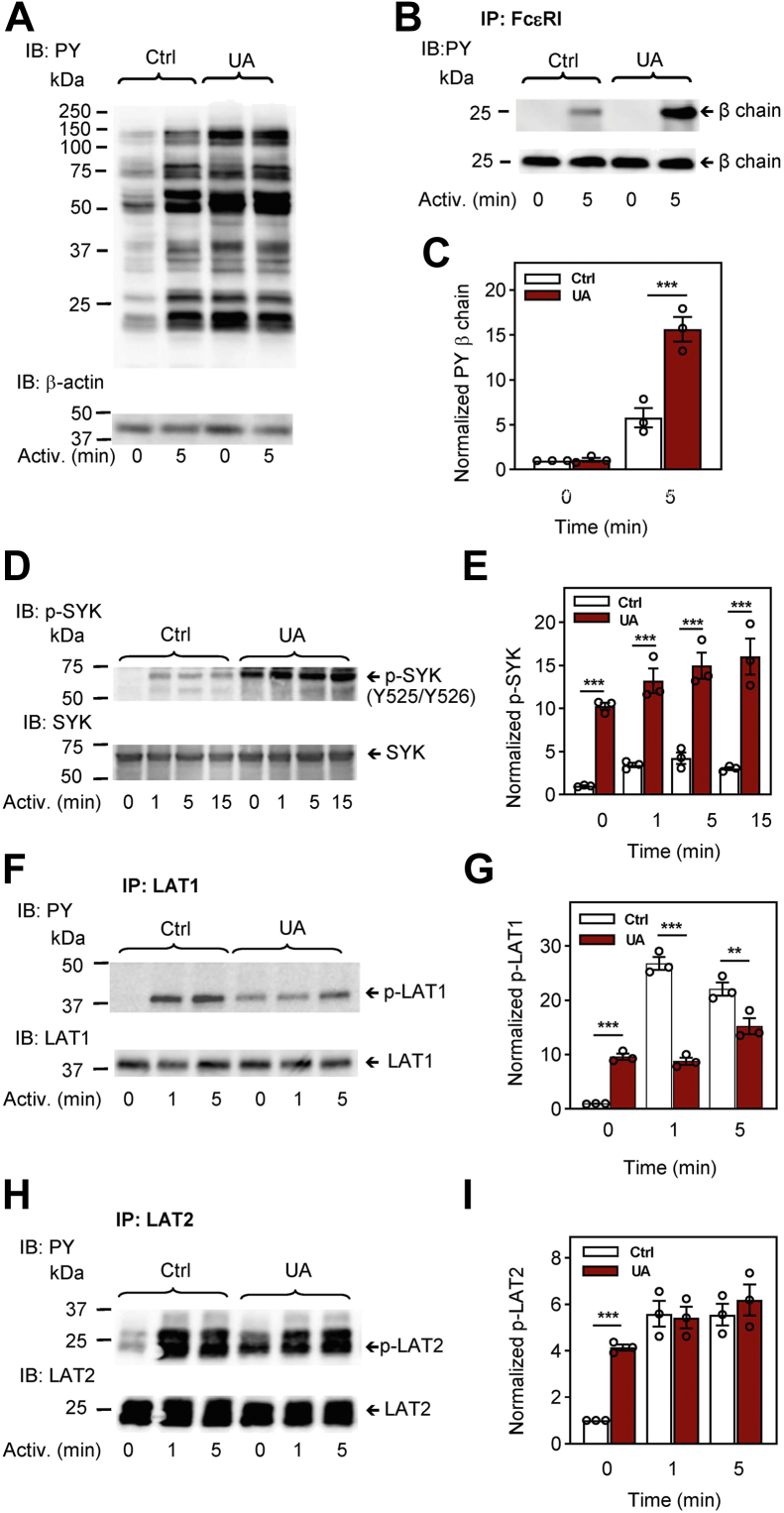
**Pretreatment with UA interferes with tyrosine phosphorylation of proteins involved in the earliest stages of FcεRI signaling, the FcεRI β subunit, SYK, LAT1, and LAT2.** IgE-sensitized BMMCs were preincubated for 15 min with vehicle (0.1% DMSO; Ctrl) or UA (50 μM) and then activated or not for the indicated time intervals with antigen (TNP-BSA; 250 ng/ml). *A*, whole-cell lysates were analyzed by immunoblotting (IB) with a phosphotyrosine-specific antibody (PY-20)-HRP conjugate (PY) for total protein tyrosine phosphorylation. Numbers on the *left* indicate the positions of molecular weight markers in kDa. For loading control, the blot was developed with an actin-specific antibody. *B* and *C*, FcεRI complexes from the cells treated as in (*A*) were immunoprecipitated (IP) and analyzed by immunoblotting with the PY-20-HRP conjugate. For loading controls, the blots were stripped and developed with the FcεRI β subunit-specific antibody. Position of the FcεRI β subunit is indicated. A representative immunoblot from three prepared in independent experiments is shown (*B*). Densitometry analysis of the immunoblots as from panel (*B*) in which signals from tyrosine-phosphorylated FcεRI β subunit in activated cells were normalized to the signals from nonactivated cells and loading control protein, the FcεRI β subunit (*C*). *D* and *E*, whole-cell lysates from cells treated for 15 min with vehicle (Ctrl) or 50 μM UA and activated with antigen (TNP-BSA; 250 ng/ml) for various time intervals were analyzed by immunoblotting for tyrosine phosphorylation of SYK (p-SYK^Y519/Y520^). *F*–*I*, BMMCs were treated as in (*D*). LAT1 (*F* and *G*) and LAT2 (*H* and *I*) were immunoprecipitated with the corresponding antibodies. The immunoprecipitates were analyzed with PY-20-HRP conjugates and protein-specific antibodies. Representative immunoblots for each phosphorylated protein with the corresponding loading controls are shown (*D*, *F*, and *H*). The results in (*E*, *G*, and *I*) show densitometry analyses of the corresponding immunoblots in which signals from tyrosine-phosphorylated proteins in activated cells are normalized to the signals in nonactivated cells and loading control proteins. Means ± SEMs in (*C*, *E*, *G*, and *I*) were calculated from three to five independent experiments. Statistical significance of intergroup differences is also shown. BMMC, bone marrow-derived mast cells; BSA, bovine serum albumin; DMSO, dimethyl sulfoxide; HRP, horseradish peroxidase; TNP, 2,4,6-trinitrophenol; UA, ursolic acid.

The first biochemically well-defined step in FcεRI signaling is tyrosine phosphorylation of FcεRI β and γ subunits by the LYN kinase, followed by the formation of the FcεRI signalosome and enhanced phosphorylation of numerous substrates (38, 39). Next, we therefore examined tyrosine phosphorylation of proteins in nonactivated or antigen-activated cells pretreated with UA or vehicle. First, we analyzed global tyrosine-phosphorylated proteins in cell lysates size-fractionated by SDS-PAGE, followed by immunoblotting with a phosphotyrosine-specific antibody PY-20-horseradish peroxidase (HRP) conjugate. As expected, in the absence of UA, antigen activation resulted in increased phosphorylation of several proteins on tyrosines (Fig. 4*A*). Treatment of the cells with UA alone (without antigen activation) also induced increased tyrosine phosphorylation of a similar set of proteins. FcεRI-mediated activation of UA-treated cells did not cause a further increase in global tyrosine phosphorylation.

Next, we examined phosphorylation of individual proteins using SDS-PAGE-fractionated whole cell lysates followed by immunoblotting with phosphoprotein-specific antibodies. Alternatively, we immunoprecipitated proteins of interest and analyzed them by immunoblotting with the mAb PY-20-HRP conjugate. To determine whether UA interferes with tyrosine phosphorylation of the FcεRI β subunit, we immunoprecipitated FcεRI from UA- or vehicle-treated cells that were activated or not with antigen. UA had no significant effect on tyrosine phosphorylation of the FcεRI β in nonactivated cells (Fig. 4, *B* and *C*). In antigen-activated cells, tyrosine phosphorylation of FcεRI-β was significantly higher in UA-pretreated cells than in control cells (Fig. 4, *B* and *C*).

An essential protein-tyrosine kinase in the FcεRI signaling is SYK, which is phosphorylated on several residues in antigen-activated cells. Tyr^519^ and Tyr^520^ of mouse SYK (equivalent to Tyr^525/526^ of human Syk) are located in the activation loop of the kinase, and their phosphorylation is crucial for SYK functioning (40). We found that 15 min treatment of BMMCs with UA induced significantly enhanced phosphorylation of Tyr^519/520^ in both nonactivated and antigen-activated cells in all time intervals analyzed (Fig. 4, *D* and *E*). SYK kinase phosphorylates the linker for activation of T cells (LAT)1 and LAT2 adapter proteins in antigen-activated cells (41); therefore, it was not surprising to find enhanced tyrosine phosphorylation of LAT1 and LAT2 in antigen-nonactivated and UA-treated cells when compared to vehicle-treated cells. After stimulation with antigen in vehicle-treated cells, phosphorylation of LAT1 and LAT2 was increased as expected. Surprisingly, there was no further increase in LAT1 and LAT2 tyrosine phosphorylation after antigen stimulation in UA-treated cells (Fig. 4, *F*–*I*).

**Figure 5:**
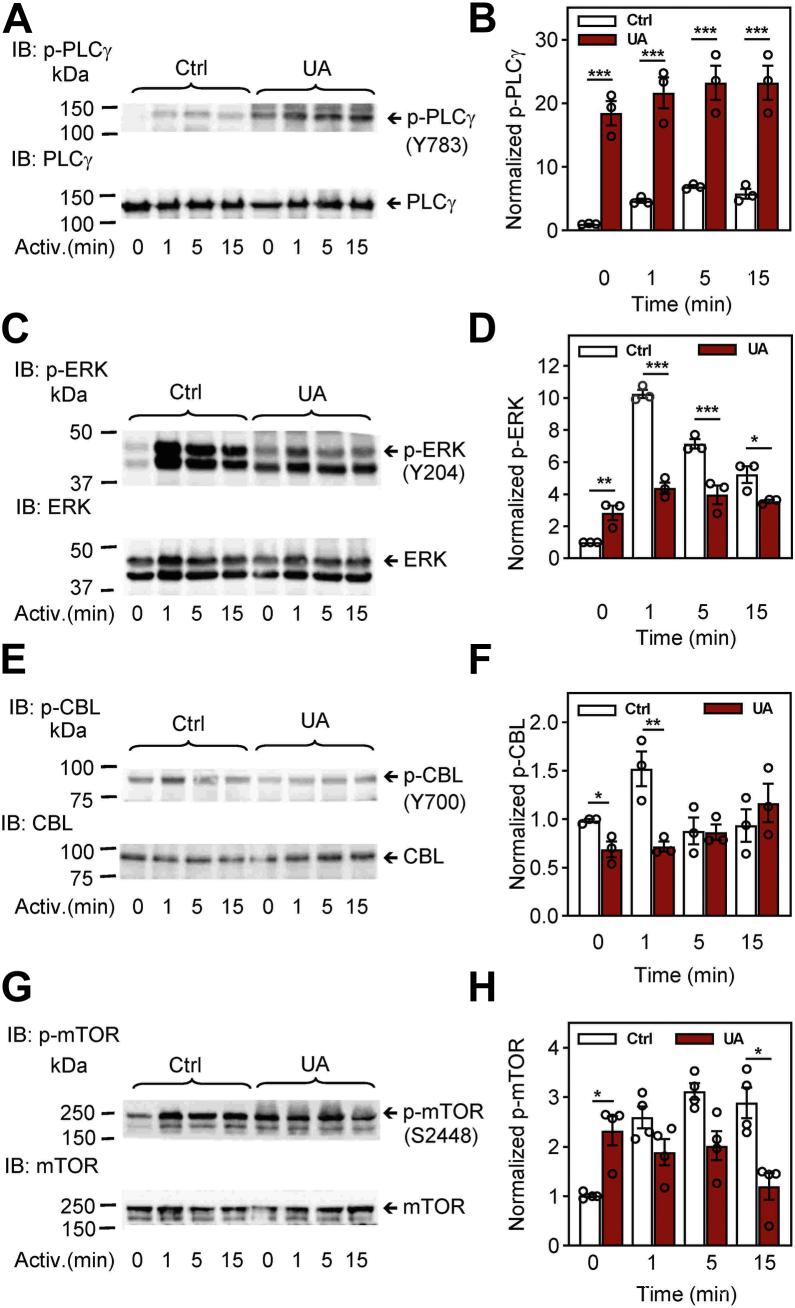
**Pretreatment with UA interferes with the phosphorylation of proteins involved in later stages of FcεRI signaling, PLCγ, ERK, CBL, and mTOR.***A*–*H*, IgE-sensitized BMMCs were preincubated for 15 min with vehicle (0.1% DMSO; Ctrl) or UA (50 μM) and then activated or not for the indicated time intervals with antigen (TNP-BSA; 250 ng/ml). Whole-cell lysates were analyzed by immunoblotting for tyrosine phosphorylation of PLCγ [p-PLCγ1^Y783^; (*A* and *B*)], ERK [p-ERK^Y204^; (*C* and *D*)], CBL [p-CBL^Y700^; (*E* and *F*)], and mTOR [p-mTOR^S2448^; (*G* and *H*)]. Representative immunoblots for each phosphorylated protein with the corresponding loading controls are shown (*A*, *C*, *E*, and *G*). The results in (*B*, *D*, *F*, and *H*) show densitometry analyses of the corresponding immunoblots in which signals from tyrosine-phosphorylated proteins in activated cells are normalized to the signals in nonactivated cells and loading control proteins. Means ± SEMs were calculated from three to four independent experiments. Statistical significance of intergroup differences is also shown. BMMC, bone marrow-derived mast cells; BSA, bovine serum albumin; DMSO, dimethyl sulfoxide; TNP, 2,4,6-trinitrophenol; UA, ursolic acid.

It is known that phosphorylation of PLCγ at Tyr^783^ by SYK kinase enhances the enzymatic activity of PLCγ (42). In agreement with enhanced phosphorylation of SYK kinase, phosphorylation of PLCγ^Tyr783^ was increased in control and antigen-activated cells treated with UA. Enhanced phosphorylation of PLCγ^Tyr783^ was highly significant in all time intervals (Fig. 5, *A* and *B*).

A critical signal transduction molecule involved in the activation of many transcription factors is ERK. To determine whether UA is involved in the phosphorylation of ERK^Tyr204^, which correlates with ERK activation, we examined ERK phosphorylation in control and UA-treated cells. We found significantly increased phosphorylation of ERK^Tyr204^ in UA-treated nonactivated cells when compared to control cells. After activation of the cells by FcεRI triggering, ERK^Tyr204^ phosphorylation was significantly lower in UA-treated cells than UA-untreated cells in all time intervals analyzed (Fig. 5, *C* and *D*).

We also analyzed phosphorylation of E3 ubiquitin-protein ligase, CBL, and mammalian target of rapamycin (mTOR) proteins. The tyrosine-phosphorylated CBL protein is involved in the negative regulation of FcεRI-mediated signaling by promoting ubiquitination of the activated receptor subunits and associated protein tyrosine kinases (43, 44). Pretreatment of BMMCs with 50 μM UA reduced phosphorylation of CBL^Tyr700^ in both nonactivated cells and cells activated with antigen for 1 min. Cells activated for 5 or 15 min and treated or not with UA showed similar phosphorylation of CBL^Tyr700^ (Fig. 5, *E* and *F*). mTOR is a serine/threonine-protein kinase, which is phosphorylated on Ser^2448^ *via* the PI3 kinase/Akt signaling pathway and regulates mast cell degranulation, cytokine production, and migration (45, 46, 47). We found significantly higher levels of phosphorylated mTOR^S2448^ in nonactivated UA-treated cells than in control nonactivated cells. After stimulation with antigen, tyrosine phosphorylation was lower in UA-treated cells than in cells treated with vehicle (Fig. 5, *G* and *H*).

Finally, we examined the phosphorylation of the LYN kinase. The catalytic activity of this enzyme is regulated in mice by phosphorylation/dephosphorylation of two conserved tyrosines at positions 397 and 508 (48, 49, 50). Phosphorylation at Tyr^397^ stabilizes the activation loop of the catalytic domain and increases LYN activity. In contrast, phosphorylation of Tyr^508^ at the C terminus of LYN by C-terminal Src kinase (CSK) stabilizes the inactive conformation of the catalytic domain. We found similar levels of LYN with phosphorylated Tyr^397^ and Tyr^508^ in UA-treated and control cells and no significant changes in phosphorylation of these regulatory tyrosines in antigen-activated cells (Fig. S1, *A*–*C*). The data suggest that the observed changes in phosphorylation of the FcεRI β subunit (Fig. 4, *B* and *C*) is not due to the enhanced activity of the LYN kinase. Rather, UA-induced changes in the spatial arrangement of plasma membrane molecules and their mobility could be involved.

### Short-term exposure to UA induces changes in the properties of detergent-resistant membrane domains

Previous studies with mast cells showed that acute cholesterol lowering or changes in the plasma membrane lipid composition resulted in changes in the properties of detergent-resistant membranes (DRMs) (35, 51, 52, 53). In further experiments, we examined whether UA induces changes in the distribution of tyrosine-phosphorylated proteins in DRMs. There is a relatively small fraction of cellular proteins, such as transmembrane adapter proteins (phosphoprotein associated with glycosphingolipid-enriched microdomains (PAG), LAT1, and LAT2) and LYN kinase, which are localized in DRMs (41, 54). In nonactivated BMMCs, these proteins exhibit tyrosine phosphorylation at relatively low levels (LAT1, LAT2) or high levels (PAG, LYN; Fig. S2, *A* and *E*). Exposure of the cells to UA resulted in an increased fraction of tyrosine-phosphorylated LYN, LAT1, and LAT2 in DRMs (Fig. S2, *B* and *E*). Activation with antigen in control cells reduced the fraction of phosphorylated LYN and PAG in DRMs (Fig. S2, *C* and *E*), as expected (55, 56). In cells pretreated with UA and activated with antigen, we observed DRMs with significantly increased levels of phosphorylated LAT1 and LAT2 (Fig. S2, *D* and *E*). These data indicate that UA interferes with the distribution of tyrosine-phosphorylated proteins in DRMs.

**Figure 6:**
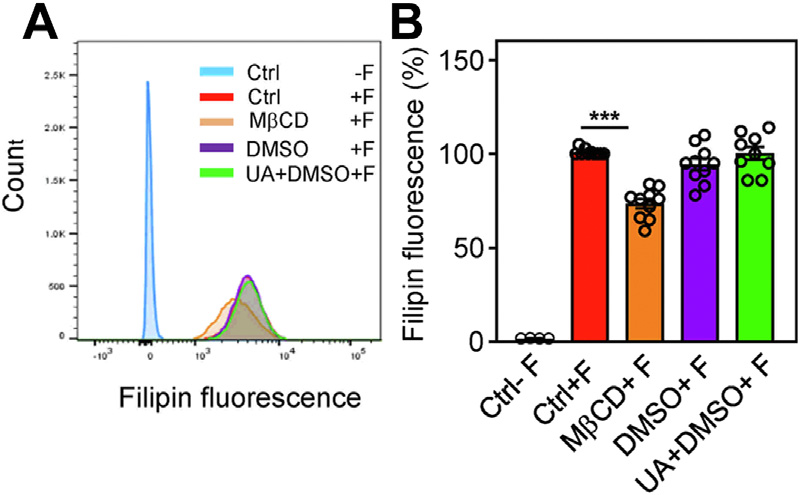
**UA does not interfere with the surface expression of cholesterol.***A*, BMMCs were untreated (Ctrl) or treated with vehicle (0.1% DMSO) or 50 μM UA in 0.1% DMSO. As a positive control, the cells were treated with 20 mM MβCD. After 30 min, the cells were washed, fixed, and stained (+F) or not (−F) with filipin. The fluorescence of the cells was evaluated by flow cytometry. Typical flow cytometry profiles are shown. *B*, data obtained as in (*A*) were normalized to fluorescence of untreated cells stained with filipin. Means ± SEMs were calculated from 10 independent experiments in each group. Statistical significance of the intergroup differences is also shown. BMMC, bone marrow-derived mast cells; DMSO, dimethyl sulfoxide; MβCD, methyl-β-cyclodextrin.

A previous study identified UA as a cholesterol-lowering drug (57). To determine whether short-term treatment of the cells to UA interferes with surface cholesterol exposure, we used filipin as a probe (58) to quantify plasma membrane surface cholesterol. In the absence of filipin, BMMCs exhibited only weak autofluorescence (Fig. 6, *A* and *B*; Ctrl-F). In the presence of filipin, the cells showed fluorescence that was similar to the control cells (Ctrl+F), cells pretreated with vehicle (0.1% DMSO+F), or containing 50 μM UA and 0.1 % DMSO. As a control, we also included cells pretreated with methyl-β-cyclodextrin (MβCD), which removes cholesterol from the cells and disrupts detergent-insoluble glycolipid domains (51, 59, 60). We found that MβCD-treated cells exhibited significantly reduced filipin fluorescence.

### Reduced patches formation of aggregated Fc**ε**RI and Thy-1 in UA-treated cells

**Figure 7:**
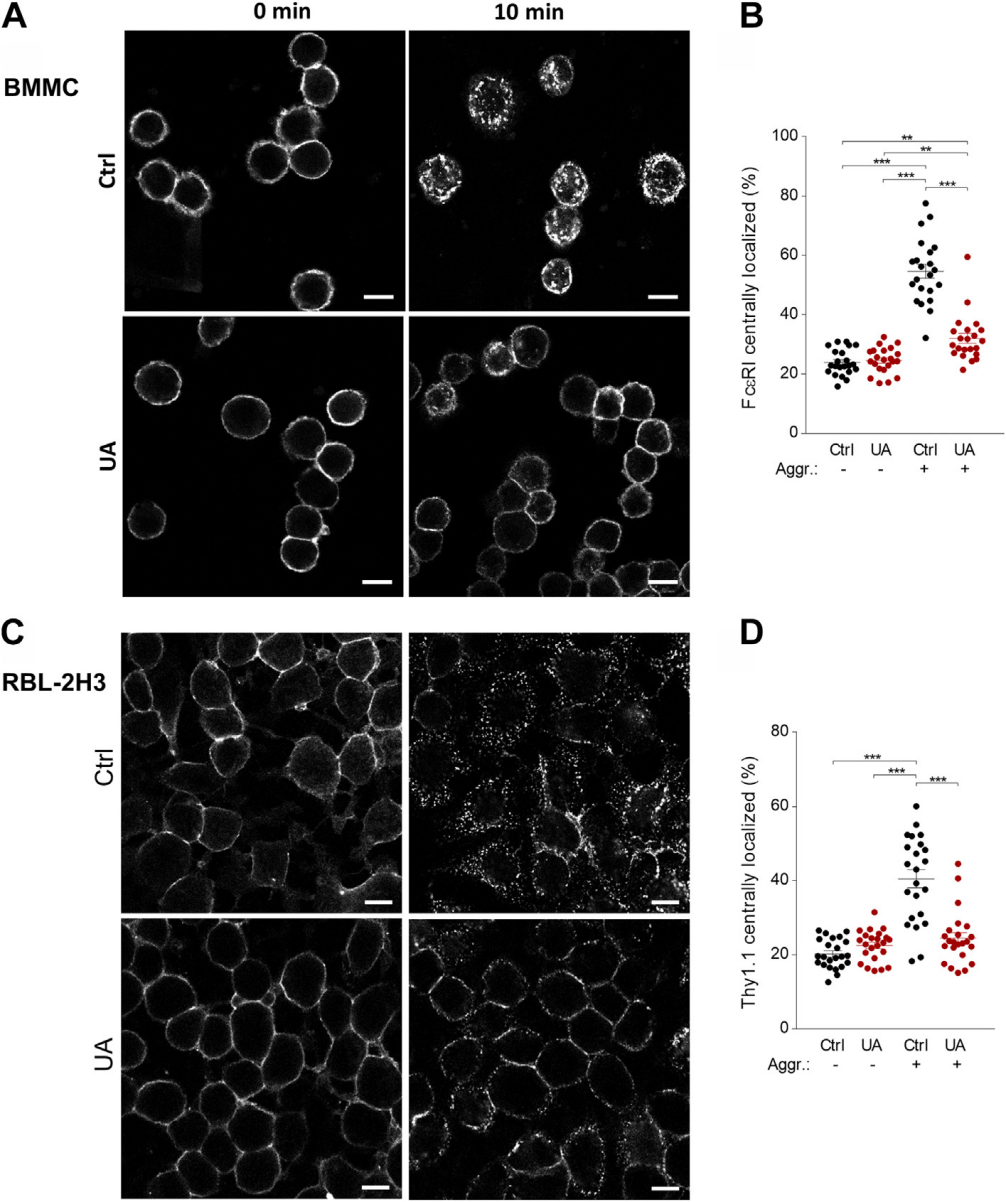
**UA interferes with the patches formation after FcεRI and Thy-1.1. crosslinking.***A* and *B*, formation of FcεRI patches. *A*, IgE-sensitized BMMCs were washed and exposed for 15 min to 50 μM UA or vehicle (Ctrl). Then, the cells were exposed to AF 488-conjugated secondary antibody for 10 min at 37 °C in the presence of 50 μM UA or vehicle (Ctrl), followed by fixing with 4% paraformaldehyde. Alternatively, the cells were fixed before exposure to AF 488-conjugated secondary antibody for 10 min. The fluorescence of cells was examined by confocal microscopy. *B*, the distribution of FcεRI in individual cells was evaluated, and the fraction of fluorescence detected centrally was calculated. Means ± SEMs and statistical significance of the intergroup differences are presented. *C* and *D*, formation of Thy-1.1 aggregates. *C*, adherent RBL-2H3 cells were treated with anti-Thy-1.1 antibody. After 30 min, the antibody was washed out, and the cells were exposed to 50 μM UA or vehicle as above. Then, the cells were fixed before (0 min) or after (10 min) exposure to AF 488-labeled secondary for 10 min at 37 °C. The fluorescence of the cells was examined by confocal microscopy. *D*, the distribution of Thy-1.1 in individual cells and were evaluated as in (*B*). Means and SEMs in (*B* and *D*) were calculate from 25 cells in three independent experiments. Statistical significance of the intergroup differences is also indicated. Bar = 10 μm. AF, Alexa fluor; UA, ursolic acid.

In our previous study, we found that pretreatment of RBL-2H3 cells with MβCD, which removes plasma membrane cholesterol, resulted in a transient increase in tyrosine phosphorylation of several proteins, increased antigen-induced and spontaneous degranulation, and increased plasma membrane mobility of the antibody-aggregated glycosylphosphatidyl-inositol–anchored protein Thy-1.1 (52). In further experiments, we examined the effect of UA on the plasma membrane mobility of the antibody-aggregated FcεRI and Thy-1.1 glycoprotein in, respectively, BMMCs and RBL-2H3 cells. The data show that pretreatment of BMMCs with UA reduced the mobility and formation of patches of aggregated FcεRI (Fig. 7, *A* and *B*). Reduced mobility by UA was also observed when antibody-mediated aggregation of the Thy-1.1 glycoprotein was analyzed (Fig. 7, *C* and *D*).

### Reduced mobility of Fc**ε**RI and cholesterol in UA-treated cells

**Figure 8:**
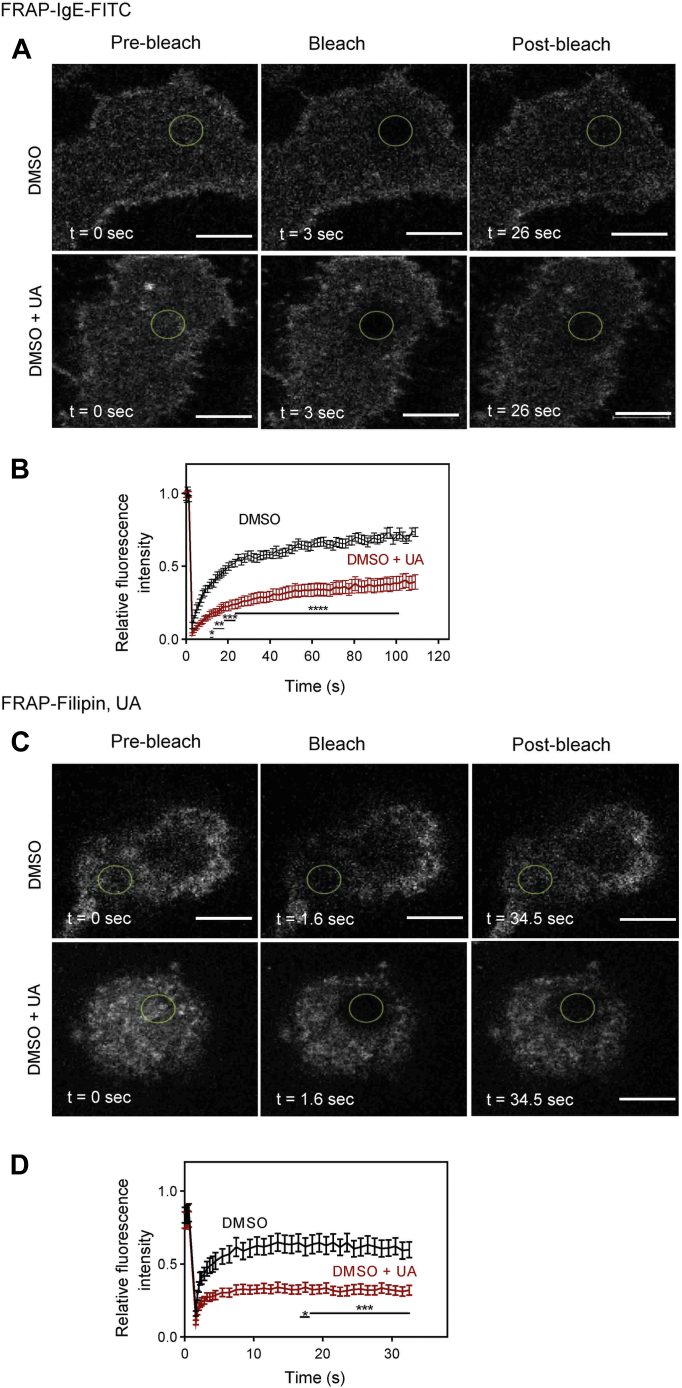
**UA inhibits the mobility of the FcεRI and cholesterol, as determined by FRAP.***A* and *B*, mobility of the FcεRI-IgE-FITC complexes. *A*, RBL-2H3 cells were exposed for 15 min to 50 μM UA in DMSO (UA) or the corresponding concentration of vehicle (Ctrl). Then the cells were exposed to IgE-FITC complexes. Image sequences are from cells at the indicated time points before photobleaching (Pre-bleach) at photobleaching (Bleach) or after photobleaching (Post-bleach; the *circled areas*). The graph in (*B*) quantifies the recovery into the photobleached areas. Means ± SEMs were calculated from 18 cells in two independent experiments with similar results. *C* and *D*, mobility of the cholesterol–Filipin complexes. *C*, RBL-2H3 cells were exposed for 15 min to 50 μM UA in DMSO or the corresponding concentration of vehicle (Ctrl). Then the cells were exposed to filipin at 50 μg/ml for 10 min. After washing, the image sequences from cells at the indicated time points before, at, or after photobleaching of the *circled areas*. The graph in (*D*) quantifies the recovery into the photobleached region. Means ± SEMs were calculated from 15 cells in two independent experiments with similar results. Statistical significance of differences between control (Ctrl) and UA-treated cells (UA) is also indicated. Bar = 10 μm. DMSO, dimethyl sulfoxide; UA, ursolic acid.

To quantify the effect of UA on the lateral diffusion of the FcεRI and cholesterol, the fluorescence recovery after the photobleaching (FRAP) method was used. When RBL-2H3 cells were exposed for 15 min to 50 μM UA, relative fluorescence of the FcεRI-IgE-FITC complexes in the photobleached area was significantly reduced when compared to cells pretreated with DMSO (vehicle; Fig. 8, *A* and *B*). From the FRAP data obtained, we calculated that control cells pretreated with vehicle (DMSO) showed a lower number of immobile FcεRI-IgE-FITC complexes (34.40 ± 3.22 %; mean ± SEM) when compared to cell pretreated with UA (64.00 ± 5.16 %). The intergroup difference was significant (*p* < 0.001).

Mobility of cholesterol–filipin complexes was also reduced in UA-pretreated cells (Fig. 8, *C* and *D*). In control cells, the immobile fraction was lower (43.00 ± 6.47 %) than in UA-pretreated cells (73.20 ± 2.93 %).

## Discussion

Data presented in this study show that short-term exposure of BMMCs to UA interferes with antigen-mediated effector functions. To understand the molecular mechanisms of the effect of UA on mast cell activation events, we examined various signaling pathways and targets. Several lines of evidence suggest that UA interferes with the function of the FcεRI-signalosome and support the lipid-centric theory of UA action at least in the earliest stages of mast cell activation.

Pretreatment of mast cells with UA in the concentration range of 10 μM to 75 μM inhibited the release of β-glucuronidase after FcεRI triggering in a dose-dependent manner. In this dose range, no increase in the spontaneous release of β-glucuronidase was observed, suggesting that UA had no toxic effect. These data are seemingly in contrast with the results of experiments showing that 15 min exposure of BMMCs to UA at 50 μM and even more at 75 μM, resulted in increased surface expression of phosphatidylserine as detected with annexin V-FITC and increased staining with PI. Phosphatidylserine is usually restricted to the inner leaflet of the plasma membrane, and this asymmetry is maintained by the activity of energy-dependent flippases and floppases, which mediate, respectively, inward-directed and outward-directed transfer of phospholipids (61, 62, 63). The equilibration of phospholipids between the two plasma membrane leaflets seems to be regulated by lipid scramblase, which facilitates the bidirectional migration of phospholipids across the lipid bilayers (64, 65). Flippases, floppases, and scramblases are transmembrane proteins, and therefore, their activity could be affected by the insertion of UA into the plasma membrane. Enhanced staining with PI could also be related to UA-induced changes in the plasma membrane properties rather than cell death. Deitch *et al*. have shown that PI does not bind only to dsDNA in the cell nucleus but also to dsRNA (66), and this could lead to an increased number of false-positive events that are associated with PI staining of RNA within the cytoplasmic compartment (32). Thus, changes in the plasma membrane properties caused by incorporating hydrophobic UA into the plasma membrane could be responsible for increased staining of cells with PI.

Detailed analysis of protein tyrosine phosphorylation in nonactivated and antigen-activated cells showed that UA dramatically affected early activation events. Interestingly, several proteins, including SYK, LAT1, LAT2, PLCγ, ERK, and mTOR, showed increased tyrosine phosphorylation induced by 15 min treatment with UA alone. These proteins are rapidly activated after triggering the cells with antigen and belong to the so-called FcεRI signalosome (39, 67). Interestingly, after stimulation with antigen, UA-treated cells, when compared to vehicle-treated cells, exhibited significantly enhanced or reduced tyrosine phosphorylation of some of these signaling proteins. One of the first well-defined enzymatic steps in antigen-activated mast cells is LYN kinase-mediated tyrosine phosphorylation of the FcεRI β subunit (68, 69). UA alone did not affect phosphorylation of this substrate but significantly enhanced phosphorylation of the FcεRI β subunit after antigen-mediated triggering. The FcεRI β subunit is phosphorylated by LYN kinase, which showed no significant changes in phosphorylation of Tyr at positions 397 and 508, serving, respectively, as a positive and a negative regulator of the LYN kinase activity. The second well-defined step in FcεRI signaling is the binding of SYK to the FcεRI γ subunit and SYK activation by its phosphorylation, followed by phosphorylation of numerous SYK substrates (70, 71). We found enhanced SYK phosphorylation in cells exposed to UA alone, and this phosphorylation was further increased in antigen-activated cells.

Enhanced phosphorylation of SYK in cells treated with UA alone was connected with increased phosphorylation of LAT1, LAT2, and PLCγ, important substrates of SYK. However, the higher phosphorylation of PLCγ was not accompanied by an increased calcium response. In fact, the levels of [Ca^2+^]_i_ were reduced in UA-pretreated and antigen-activated cells, which was likely responsible for impaired degranulation. The lower [Ca^2+^]_i_ in UA-treated and FcεRI-activated cells was mainly caused by reduced calcium uptake, as determined in experiments measuring ^45^Ca uptake. These data were supported by reduced [Ca^2+^]_i_ after adding Ca^2+^ to cells activated by antigen in Ca^2+^-free buffered salt solution (BSS). Similar inhibition of degranulation and calcium response was observed in UA-treated cells activated by thapsigargin. Since thapsigargin bypasses all early FcεRI-mediated signaling steps, it is likely that a key factor leading to UA-mediated inhibition of degranulation in antigen-activated cells is due to reduced Ca^2+^ uptake. The negative effect of UA on calcium channel activity could be related to UA-mediated changes in lipid bilayer properties (72). In fact, previous studies showed that compounds that increase rigidity (73) or reduce fluidity (74) of the plasma membrane inhibit store-operated Ca^2+^ (SOC) channels. Insertion of UA into the plasma membrane could inhibit SOC channels through direct interaction of UA with Orai1 protein in a similar way as was described for inhibition of Orai1 by cholesterol (75).

It should be noted that UA negatively regulated only some of the activation events. Thus, UA-treated cells exhibited reduced chemotaxis toward antigen but not toward PGE_2_ or SCF. Although the molecular basis of these differences is not known, it could be, at least in part, dependent on UA-mediated changes in the plasma membrane properties. UA is a multicyclic rigid planar structure with a hydrophilic 3β-hydroxyl group. In its structure, UA is similar to cholesterol, which is also a multicyclic rigid planar structure with a hydrophilic 3β-hydroxyl group and a hydrophobic side chain. Thus, both UA and cholesterol can tightly assemble the molecules of the phospholipid bilayer and cause the membrane to be less fluid (76, 77). Furthermore, regarding its cell membrane–associated functions, cholesterol is also implicated in the modulation of cellular signal transmission and intracellular trafficking by contributing to lipid raft assemblies (78, 79, 80). Comparable properties of cholesterol and UA in their fluidity-modulating and condensing effects were also shown in studies using liposomal membranes (27). In these studies, UA was shown to locate near the surface in the vicinity of the phospholipid headgroups, with orientation depending on the lipid composition. In complex lipid systems, UA interacted specifically with POPE (1-palmitoyl-2-oleoyl-sn-glycero-3-phosphoethanolamine), PSM (N-palmitoyl-D-erythro-sphingosyl-phosphorylcholine), and cholesterol and excluded POPG (1-palmitoyl-2-oleoyl-sn-glycero-3-phospho-(1′-rac-glycerol) from its surrounding (28, 31). Furthermore, UA reacted specifically with phosphatidylethanolamine phospholipids through hydrogen bonding, disorganizing the membrane interface and thus disrupting the membrane integrity (81). The combined data suggest that UA, similarly to other triterpenoids and hydrophobic bioactive molecules, incorporates into biological membranes and modulates their fluidity, morphology, and permeability (29, 82). Based on the similarity of cholesterol and UA, it is not surprising that chemotaxis of mast cells toward antigen and PGE_2_ showed opposite effects when the cells were pretreated with UA or MβCD (83, 84). The similarity between UA and cholesterol could also explain the increased levels of TNF-α in antigen-activated mast cells pretreated with UA (this study) or cells activated by cholesterol with ionomycin (85, 86). However, as shown in our study, the release of TNF-α from UA-treated cells is reduced. This could be caused by reduced [Ca^2+^]_i_, which is important for TNF-α exocytosis (87).

The activation processes are initiated in DRM microdomains called lipid rafts. In nonactivated cells, pretreatment with UA for 15 min resulted in an increased amount of tyrosine-phosphorylated LYN, LAT1, and LAT2 in DRMs. In antigen-activated cells, pretreatment with UA resulted in an enhanced amount of tyrosine-phosphorylated LYN and LAT2 in DRMs. These data suggest that the properties of lipid microdomains are disorganized by UA, which could have contributed to the observed changes in the signaling properties of the FcεRI signalosome.

In experiments in which we followed the formation of patches of aggregated FcεRI and Thy-1.1 glycoprotein, we obtained evidence that UA reduced aggregation of the surface components. In connection with these experiments, it is relevant that lowering plasma membrane cholesterol with MβCD resulted in acceleration of Thy-1 aggregation and rapid formation of caps (88). Direct evidence that mobility of plasma membrane components is reduced by UA was obtained in FRAP experiments in which UA significantly increased the immobile fraction of FcεRI and cholesterol.

In most of the experiments in this study, we used UA at a concentration of 50 μM. Although this is a relatively high concentration, it can be clinically relevant when the drug is administered topically. In the mouse model, oral administration of UA suppressed both IgE-mediated passive cutaneous anaphylaxis and ovalbumin-induced active systemic anaphylaxis at concentrations similar to those of dexamethasone, which is clinically used (20).

## Conclusions

**Figure 9:**
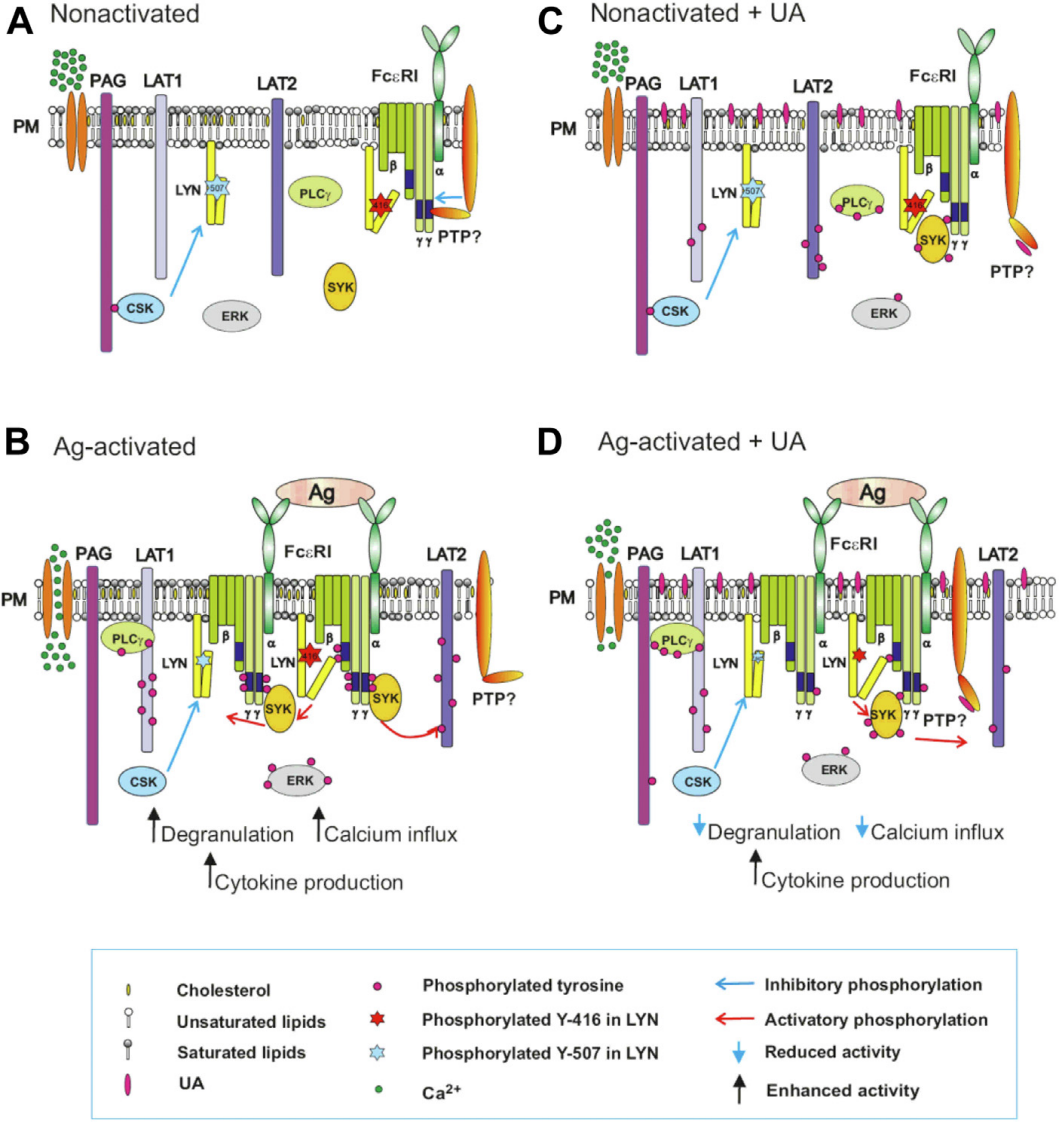
**Model of FcεRI-mediated activation in UA-treated cells.** In nonactivated cells (*A*), the topography of plasma membrane components keeps all components of the FcεRI signalosome, including kinases and phosphatase, in a steady state. *B*, antigen-induced aggregation of FcεRIs disturbs this steady state in favor of kinases, leading to tyrosine phosphorylation of numerous substrates and propagation of the signals resulting in increased degranulation, calcium response, and cytokines production. *C*, Inserting hydrophobic UA into the plasma membrane interferes with the properties of plasma membrane components, leading to changes in their mobility and increased phosphorylation of the several critical proteins of the FcεRI signalosome. UA could interfere with the topography of kinases, phosphatases, and their substrates. *D*, In UA-treated and antigen-activated cells, disturbances of the plasma membrane components cause a suboptimal performance of the FcεRI signalosome, leading to a reduced calcium response and degranulation. Interestingly, cytokine production is enhanced in UA-treated and antigen-activated cells but the secretion of the cytokines is diminished. UA, ursolic acid.

As depicted in Figure 9, there is a coordinated synergy of lipid-based and protein-based interactions in the earliest stages of FcεRI signaling. In nonactivated cells, a large fraction of LYN kinase preferentially partitions into liquid-order (Lo)-like lipidic nanodomains in the plasma membrane due to its saturated lipid anchor (palmitoyl and myristoyl). Lo-like domains also carry other components of the FcεRI signalosome, such as transmembrane adapter proteins LAT1, LAT2, and PAG. IgE receptors seem to be separated into liquid-disordered (Ld)-like nanodomains, where phosphatases keep phosphorylation of the FcεRI subunits at nonfunctional thresholds (39, 41, 89). Activation of IgE-sensitized cells by multivalent antigen leads to aggregation of antigen-IgE-FcεRI complexes, the enhanced association of the FcεRI aggregates with Lo-like domains, and reduced physical and functional association of the phosphatases with the FcεRI complexes. These changes alter the balance between kinases and phosphatases in favor of kinases, leading to increased phosphorylation of the FcεRI subunits (39). The phosphorylated FcεRI γ subunit is a transient anchor of SYK kinase that phosphorylates adapter proteins and contributes to further propagation of the activation signal, resulting in enhanced calcium response, degranulation, and cytokine production. UA is a hydrophobic pentacyclic triterpenoid, which incorporates into the plasma membrane and changes its properties, including mobility of the plasma membrane components. In this way, UA itself could change the balance between kinases and phosphatases, leading to tyrosine phosphorylation of numerous substrates without receptor triggering. Activation of UA-pretreated cells with antigen leads to further phosphorylation of some kinase substrates (FcεRI β subunit, SYK kinase, PLCγ) or reduced phosphorylation of other signaling proteins (LAT1, ERK, CBL, mTOR). UA-dysregulated signaling events could lead to a reduced calcium response and degranulation or increased production of cytokines. Although UA could bind to and modify some signaling proteins, the earliest FcεRI-activation events affected by UA seem to reflect a direct interaction of UA with the plasma membrane lipids. The combined data suggest that UA induces plasma membrane perturbations by directly interacting with the plasma membrane lipids and support the lipid-centric hypothesis of UA action at the initial stages of mast cell activation.

## Experimental procedures

### Antibodies and reagents

The following mAbs were used: mouse IgE mAb specific for 2,4,6-trinitrophenol (TNP), clone IGEL b4.1 (90), anti-FcεRI β subunit, clone JRK (91), anti-SYK (92), anti-LAT2 (NAP-07) (93), anti-LAT1 (94), and anti-Thy-1.1 (95). Polyclonal antibodies specific for SYK, LAT1, LAT2, and IgE were prepared by immunization of rabbits with the corresponding recombinant proteins or their fragments (54). Anti-IgE was prepared by immunization rabbits with IGEL b4.1 mAb. Polyclonal antibodies specific for phospholipase C (PLC) γ1 (1249, catalog no.: #sc-81, RRID: AB-632202), phospho-PLCγ1^Y783^(catalog no.: #sc-12943, RRID: AB-654725), ERK1 (C-16, catalog no.: #sc-93, RRID: AB-631453), phosho-ERK^Y204^ (catalog no.: #sc-7976, RRID: AB-2297323), CBL (C-15, catalog no.: #sc-170, RRID: AB-2259627), phosho-CBL^Y700^ (catalog no.: #sc-26140, RRID: AB-2070464), actin (H-300, catalog no.: #sc-10731), as well as HRP-conjugated goat antimouse IgG (catalog no.: #sc-2005, RRID: ABbin631736) and goat anti-rabbit IgG (catalog no.: #sc-2004, RRDI: 631,746) were obtained from Santa Cruz Biotechnology Inc antibodies specific for phospho-SYK ^Y525/Y526^ (corresponding to mouse SYK^Y519/Y520^; C87C1, catalog no.: #2710, RRID: AB-2197222), m-TOR (catalog no.: #2972S, RRID: AB - 330978), phospho-mTOR ^S2448^ (catalog no.: #2971S, RRID: AB-330970), phospho-SFK^Y416^ (mouse LYN^Y397^; catalog no.: #2101) phospho-LynY507 (mouse LYN^Y508^; catalog no.: #2731) were obtained from Cell Signaling. HRP-conjugated anti-phospho-tyrosine-specific mAb (PY-20; catalog no.: #610,012, RRID: AB-11204229) was purchased from BD Biosciences. Mouse-specific TNF-α-conjugated to R-phycoerythrin (catalog no.: #561063) was from BD Pharmingen. TNP-bovine serum albumin (BSA) conjugate (15–25 mol TNP/mol BSA) was produced as described (96). Thapsigargin was obtained from Invitrogen, and recombinant murine SCF and IL-3 were from PeproTech EC. ^45^Ca (specific activity, 773 MBq/mg Ca^2+^) was purchased from the Institute of Isotopes Co, Ltd Fura-2 acetoxymethyl ester (Fura-2-AM) and Alexa Fluor (AF) 488–conjugated donkey antimouse IgG (catalog no.: #A-21202, cross-reacting with mouse IgE) were from Life Technologies. RT-PCR reagents were obtained from Top-Bio. Annexin V-FITC (EXB0024), annexin-binding buffer (10×, EXB0019), and PI (EXB0018) were purchased from EXBIO. UA (U6753), saponin (47036), filipin (F9765), DMSO (D8418), 4-methylumbelliferyl-β-D-glucuronide hydrate (M9130), MβCD (332615), and all other reagents were from Sigma–Aldrich.

### Mice and cells

Mice were bred and maintained in a specific pathogen-free facility of the Institute of Molecular Genetics, used in compliance with the Institute guidelines, and approved by the Institute Commission for Animal Welfare and Protection (Approval No 23/2013). BMMC precursors were isolated from the femurs and tibias of 6- to 8-week-old mice of the C57BL/6 genotype. They were cultured for 8 to 12 weeks in a complete growth medium consisting of RPMI-1640 medium supplemented with 100 U/ml penicillin, 100 μg/ml streptomycin, 71 μM 2-mercaptoethanol, minimal essential medium, nonessential amino acids, 0.7 mM sodium pyruvate, 2.5 mM L-glutamine, 12 mM D-glucose, recombinant mouse SCF (15 ng/ml), recombinant mouse IL-3 (15 ng/ml), and 10% (vol/vol) fetal calf serum. In experiments presented in Figs. 3 and 7, we used cells of the BMMC line (BMMCL) (97), which were cultured under the same conditions as BMMCs, except that SCF was omitted from the culture medium. For experiments presented in Figs. 7 and 8, we also used RBL cells, clone 2H3 (98), which were cultured as previously described (99).

### Annexin V-FITC and PI staining

The annexin V–binding assay was used for quantification of phosphatidylserine surface expression in BMMCs treated with various concentrations of UA. Briefly, the cells were washed with BSS (20 mM Hepes, pH 7.4, 135 mM NaCl, 5 mM KCl, 1.8 mM CaCl_2_, 1 mM MgCl_2_, 5.6 mM glucose) supplemented with 0.1% BSA. The assay was performed on a 96-well plate. Cells (0.25 × 10^6^) in 100 μl BSS-0.1% BSA were exposed to various concentrations of UA for 15 min at 37 °C, centrifuged at 4 °C and washed with ice-cold 1× annexin-binding buffer. The cell pellets were resuspended in 50 μl of ice-cold 1× annexin V–binding buffer supplemented with 5 μl annexin V-FITC or 1× annexin V buffer alone for unstained control cells. Cells were incubated for 30 min on ice in the dark, and 5 min before flow cytometry analysis, 50 μl of the binding buffer with or without PI was added. Samples were measured in LSRII FACS DIVA (BD Bioscience). Annexin V-FITC binding was analyzed by flow cytometry using the FITC signal detector (FL1) and PI staining by the phycoerythrin emission detector (FL2).

### Cell activation, degranulation, and calcium response

BMMCs were sensitized in the complete growth medium without IL-3 and SCF, supplemented with IgE (1000× diluted ascites formed by hybridoma cells IGEL b4.1). After 12 to 14 h of incubation, cells were washed and resuspended in BSS-0.1% BSA at a concentration of 1.5 × 10^6^ cells/ml. Cells (100 μl aliquots) were transferred into wells of a 96-well cell culture plate. The cells were pretreated for 15 min with various concentrations of UA or vehicle (0.1% DMSO) in BSS-0.1% BSA added in 100 μl aliquots and challenged with 20 μl of antigen (TNP-BSA conjugate) at a concentration of 250 ng/ml. When the UA-treated cells were activated by thapsigargin, the sensitization step was omitted. The degree of degranulation was determined as the amount of β-glucuronidase released into the supernatants as described (56) using 4-methylumbelliferyl-β-D-glucuronide hydrate as a substrate. For the total amount of β-glucuronidase, the cells were treated with 0.5% Triton X-100. Fluorescence was determined by an Infinite M200 microtiter plate reader (Tecan) at 355-nm excitation and 460-nm emission filters.

Changes in the [Ca^2+^]_i_ were analyzed using cytoplasmic reporter Fura-2-AM as described (56). Briefly, IgE-sensitized cells were incubated with Fura-2-AM in BSS-0.1% BSA supplemented with 2.5 mM probenecid (35). After 30 min, cells were washed and treated with UA or vehicle in the presence of probenecid for 15 min. Before the measurement, the cells were washed and resuspended in BSS-0.1% BSA without probenecid. The free cytoplasmic Ca^2+^ levels were determined by an Infinite M200 microtiter plate reader in a NUNC white 96-well plate (Thermo Scientific) with excitation wavelengths at 340 and 380 nm and constant emission at 510 nm. To determine the effect of UA on antigen-induced changes in [Ca^2+^]_i_ in the absence of extracellular calcium, the same method was used, except that BSS without CaCl_2_ was used, and after antigen triggering, CaCl_2_ was added as indicated in Figure 2*D*.

Extracellular calcium uptake was measured as previously described (53). Briefly, IgE-sensitized or nonsensitized cells (2 × 10^6^) were washed in BSS-0.1% BSA, resuspended in 100 μl BSS-0.1% BSA with 1 mM Ca^2+^, and mixed with 100 μl UA. After 15 min incubation at 37 °C, the cells were activated for 10 min at 37 °C by adding 20 μl BSS-0.1% BSA supplemented with a mix of ^45^Ca^2+^ and antigen (250 ng/ml; IgE-sensitized cells) or thapsigargin (1 μM; IgE nonsensitized cells). The reaction was terminated by placing the tubes on ice, and cells with bound ^45^Ca^2+^ were separated from free ^45^Ca^2+^ by centrifugation at 1200*g* for 15 min at 4 °C through 12% BSA as described (100). Cell pellets were recovered by freezing the tubes, slicing off the tube bottom, and solubilizing the cells with 1 ml of 1% Triton X-100. The radioactivity of the samples was measured in 10 ml scintillation liquid (EcoLite; ICN Biomedicals) using a scintillation counter with QuantaSmart software (PerkinElmer).

### Cell chemotaxis

Chemotaxis responses were assayed in a 24-well transwell chamber (Corning) with 8 μm pore size polycarbonate filters in the upper wells as previously described (56). TNP-specific IgE-sensitized BMMCs or nonsensitized cells were pretreated or not with UA (50 μM) for 15 min at 37 °C, and 0.3 × 10^6^ cells in 120 μl of chemotaxis medium (RPMI-1640 supplemented with 1% BSA and 20 mM Hepes pH 7.4) were added into the transwell insert. Chemoattractants (antigen (250 ng/ml), PGE_2_ (100 nM), or SCF (50 ng/ml)) were added to the lower compartments in 0.6 ml aliquots of chemotaxis medium. After 6 h incubation (37 °C, 5% CO_2_ in air), cells migrating into lower compartments were counted in 50 μl aliquots using the Accuri C6 flow cytometer (BD Bioscience).

### Cell lysates, immunoprecipitation, and immunoblotting

IgE-sensitized cells were treated or not with UA for 15 min at 37 °C, followed by activation of the cells with antigen (TNP-BSA, 250 ng/ml) for different time intervals. Toward the end of the activation period, the cells were centrifuged and the pellet was resuspended in ice-cold immunoprecipitation buffer (25 mM Tris–HCl pH 8.0, 140 mM NaCl, 1 mM Na_3_VO_4_, 2 mM EDTA, 1 μg/ml aprotinin, 1 μg/ml leupeptin, and 1 mM PMSF) supplemented with 0.2% Triton X-100 (for FcεRI immunoprecipitation). Lysis buffer of the same composition but with 25 mM Tris–HCl, pH 7.5, supplemented with 1% n-dodecyl-β-D -maltoside and 1% Nonidet P-40 was used for LAT1 and LAT2 immunoprecipitations. After incubation on ice for 30 min, the lysates were centrifuged (16,000*g* for 5 min at 4 °C), and the proteins in the postnuclear supernatants were immunoprecipitated with the corresponding antibodies prebound to UltraLink-immobilized protein A (Pierce, Thermo Scientific). The immunoprecipitated proteins were size fractionated by SDS-PAGE and analyzed by immunoblotting with PY-20-HRP conjugate or with protein-specific antibodies, followed by HRP-conjugated antimouse or anti-rabbit IgG antibodies. Several tyrosine-phosphorylated proteins were quantified by direct immunoblotting of SDS-PAGE–fractionated whole cell lysates with phosphoprotein-specific antibodies. For loading controls, protein-specific antibodies were used. The primary antibodies were detected with HRP-conjugated secondary antibodies. The HRP signal was detected by Luminescent Image Analyzer LAS-3000 (Fujifilm) and quantified with AIDA software (Raytest GmbH). The levels of phosphorylated proteins were normalized to the loading controls run in parallel gels (56).

### Sucrose density gradient fractionation

Sucrose density gradient separations were performed as previously described (101). Briefly, IgE-sensitized BMMCs (15 × 10^6^) were pretreated or not with UA for 15 min and then activated or not with antigen (250 ng/ml) for 5 min. After centrifugation, the cells were lysed on ice in 0.8 ml lysis buffer (10 mM Tris–HCl, pH 8.0, 50 mM NaCl, 2 mM EDTA, 10 mM glycerophosphate, 1 mM Na_3_VO_4_, 1 mM PMSF, 0.5 U/ml aprotinin, and 0.5 U/ml leupeptin) supplemented with 1% Brij-96. The gradient was formed by adding 0.5 ml of 80% sucrose stock solution to the bottom of a polyallomer tube (13 × 51 mm; Beckman Instruments) followed by 1.5 ml of 40% sucrose containing the cell lysate, 2 ml 30%, and 1 ml 5% sucrose. After 4 h centrifugation at 4 °C (210,000*g* using an SW55 Ti rotor), 10 fractions were collected from the top of the gradient, and individual fractions were analyzed by SDS-PAGE followed by immunoblotting with PY-20-HRP and protein-specific antibody.

### Quantification of cell surface cholesterol

After 30 min incubation at 37 °C with shaking, the cells were washed and fixed for 10 min at 22 °C (in the dark) with 4% paraformaldehyde in staining buffer. The fixed cells were washed twice in staining buffer, exposed or not to 50 μl filipin (50 μg/ml PBS), and incubated for 30 min in the dark at 22 °C. After incubation, filipin was washed out twice, the cells were resuspended in staining buffer, and cholesterol levels were quantified using a BD FACSymphony flow cytometer with UV light laser (355 nm) and UV detector (450/50 nm).

### Detection of cytokine mRNAs

IgE-sensitized or nonsensitized cells of the BMMC line were pretreated with 50 μM UA or vehicle (0.1% DMSO) for 15 min and then activated or not with antigen (TNP-BSA; 250 ng/ml) for 60 min. For cytokine mRNA detection, we used the RNA-mediated oligonucleotide annealing, selection, and ligation (RASL)-quantitative PCR (qPCR) protocol, adapted from the miRNA detection method described previously (102). Briefly, RNA was isolated using a MicroElute Total RNA Kit (Omega Biotek) according to the manufacturer’s instructions. Two DNA oligonucleotide probes (A and B, see later) specific for each cytokine mRNA were designed and synthesized. The oligonucleotide probes contained 15 to 16 nucleotide sequence regions complementary to a particular mRNA and a sequence for qPCR primer annealing (19 nucleotides). One of the probes (oligonucleotide B) has a phosphate on its 5′end to enable its ligation with the second probe (oligonucleotide A). The complementary sequences of both oligonucleotides are designed in such a way that they hybridize to mRNA right next to each other and the whole sequence is intron spanning. Both pairs of oligonucleotides (10 fmol each) for the given mRNA were mixed with the RNA sample (50 ng) and hybridized in 100 mM Tris–HCl pH 7.5 and 20 mM MgCl_2_ for 2 min at 85 °C, followed by 1 min incubation steps at temperatures 5 °C lower than in the preceding step with the final step at 25 °C. Next, 10 μl of the hybridization reaction was mixed with 1.25 U of SplintR ligase (New England BioLabs), 0.14 μl of 100 mM ATP, and 4 μl of 2× concentrated TP buffer (Top-Bio) containing 15 mM Tris–HCl, pH 8.8, 40 mM (NH_4_)_2_SO_4_, 0.4 M trehalose, 2 M 1,2-propanediol, 0.02% Tween 20, and 5 mM MgCl_2_ (103). The samples were ligated for 1 h at 37 °C. After ligation, 5 μl aliquots were mixed with 15 μl qPCR Master Mix containing 6.1 μl of 2× concentrated TP buffer, 1 U of Taq DNA polymerase Unis, 0.4 μl of 10 mM dNTP mix, 0.3 μl of 10 μM primer mix, 0.1 μl of 0.1 mM SYBR Green I (10 mM stock (104) diluted 1:100 in DMSO), and 7.9 μl of PCR H_2_O. The following thermal cycling conditions were used: initial incubation at 94 °C for 3 min followed by 45 cycles, each consisting of 10 s at 94 °C, 10 s at 60 °C, and 8 s at 72 °C in RealPlex EP Mastercycler. The whole procedure was carried out in triplicates in white 96-well PCR plates. GAPDH was used as a reference gene. Oligonucleotides for TNF-α mRNA detection are as follows: TNF-α probe A: 5′- CTC GAC CTC TCT ATG GGC AAT TTT GAG AAG ATG A -3′; TNF-α probe B: 5′- (phos) TCT GAG TGT GAG GGT CGG AGA CAC GCA GGG CTT AA -3′. PCR primers for the cytokine forward primer: 5′- GCT CGA CCT CTC TAT GGG C-3′; reverse primer: 5′ –TTA AGC CCT GCG TGT CTC C-3′. Oligonucleotides for GAPDH mRNA detection are as follows: GAPDH probe A: 5′- CCC GTA ATC TTC ATA ATC CGA G TGG TGC AGG ATG CAT -3′; GAPDH probe B: 5′- (phos) TGC TGA CAA TCT TGA CTG AAT GGC ATC GAG TAC -3′. PCR primers for GAPDH: forward primer: 5′- CCC GTA ATC TTC ATA ATC CGA G -3′; reverse primer: 5′- GTA CTC GAT GCC ATT CAG -3′. The underlined probe sequences are the sites for PCR primer annealing.

### Bead-based immunoassay for quantification of TNF-α released into supernatants

BMMCs (2 × 10^6^) were sensitized with IgE, then exposed to 50 μM UA or vehicle for 15 min, followed by activation or not with 250 ng/ml of TNP-BSA for 4 h. Then, the cells were centrifuged, and the supernatants were used for TNF-α quantification using the bead-based immunoassay (LEGENDplex multianalyte flow assay; mouse B effector panel 8-plex [BioLegend; catalog no.: 740820]) according to manufacturer’s instructions. BD Symphony flow cytometer equipped with 488 and 637 nm lasers was used for data collection. The data were processed with LEGENDplex data analysis software.

### Quantification of cellular and surface TNF-α

To quantify cellular TNF-α, the IgE-sensitized BMMCs were pretreated for 15 min with 50 μM UA or vehicle and then activated with antigen (TNP-BSA). After 2.5 h, the cells were centrifuged at 805*g* for 3 min at 21 °C, followed by fixing with paraformaldehyde (4% in PBS) for 15 min at 21 °C. After washing in PBS, the cells were permeabilized with 0.1% saponin and further processed as described (105). To quantify TNF-α exposed on the plasma membrane, we omitted the permeabilization of the cells with saponin.

### Fc**ε**RI and Thy-1.1 mobility assay

For the FcεRI mobility assay, 15-well multitest microscopy slides (MP Biomedicals) were coated overnight with fibronectin (10 μg/ml in PBS), blocked with 4% BSA in PBS (15 min at 37 °C), and washed twice with PBS. IgE-sensitized BMMCs were left to attach to fibronectin-coated slides for 30 min in BSS-0.1% BSA at 37 °C. Then, the cells were exposed to 50 μM UA or vehicle (0.1% DMSO) in BSS-0.1% BSA. After 15 min incubation at 37 °C, the cells were exposed to AF 488-labeled anti-IgE for 10 min at 37 °C in the presence of 50 μM UA or vehicle and then washed and fixed with 4% paraformaldehyde. Alternatively, the cells were incubated with UA or vehicle as aforementioned, washed, fixed, and then labeled for 10 min with AF 488-labeled anti-IgE. For Thy-1.1 mobility assay, RBL-2H3 cells were grown on sterilized 8-well multitest slides (MP Biomedicals) for 18 h, washed with BSS-0.1% BSA, and treated with anti-Thy-1.1 mAb. After 30 min, the antibody was washed out and the cells were exposed for 15 min at 37 °C to 50 μM UA or vehicle. Then, the cells were exposed to AF 488-labeled anti-IgG for 10 min, washed, and fixed with 4% paraformaldehyde. As controls, anti-Thy-1.1-labeled cells were exposed to 50 μM UA or vehicle, fixed, and labeled with AF-labeled secondary antibody. The cells were washed and the samples were mounted using 90% glycerol supplemented with 5% n-propyl gallate. The mobility of FcεRI and Thy-1.1 was determined in the analytical volume comprising the apical part of the attached cells defined by pinhole size 1.5 AU. Images of the cells were acquired with a confocal laser scanning microscope Leica TCS SP5 equipped with X40/1.40 NA. oil-immersion objective. In each experiment, all images were acquired at identical microscope settings. The distribution of FcεRI and Thy-1.1 in individual cells was evaluated, and the fraction of fluorescence detected in the cell periphery (1 μm width) and centrally was calculated using ImageJ (106; https://imagej.nih.gov/ij/). Data are presented as a fraction of fluorescence localized centrally.

### FRAP analysis

FRAP measurements were performed using the Leica Stellaris 8 Falcon microscopic system equipped with an oil immersion objective (60× /1.4 NA) and an environmental chamber controlling CO_2_ concentration (5%) and temperature (37 °C). The cells were grown in glass-bottomed wells of the 96-well plate (*In Vitro* Scientific, 96101N), and the photobleaching was achieved by a 100 ms laser pulse at 488 nm with 100% of the laser power level. Fluorescence intensity was monitored in a circular area of 1 μm in diameter. Confocal images were obtained from a single optical plane corresponding to the point of contact between the cell and the glass. The obtained time lapses were analyzed using LasX software (https://www.leica-microsystems.com/products/microscope-software/p/leica-las-x-ls/). Subsequently, the background was subtracted from the intensities of the FRAP area, and curves were corrected for bleaching. The resulting FRAP curves (15–18 measurements for each experiment) were fitted individually biexponentially to obtain an immobile fraction (107, 108). All optimizations were performed in MATLAB R2021b (MathWorks), using a nonlinear least-squares fit (lsqcurvefit).

### Statistical analysis

Statistical significance of intergroup differences was determined by one-way ANOVA with Tukey’s post-test using Prism version 5.04 graphics and statistic software package (GraphPad). Data in Figure 8, *B* and *D* were calculated with one-way ANOVA with Tukey’s multiple comparison test. In experiments where only two groups were compared, the statistical significance of differences was evaluated with Student’s unpaired, two-tailed *t* test. All values are expressed as means ± SEMs. Significance levels: ∗*p* < 0.05; ∗∗*p* < 0.01; ∗∗∗*p* < 0.001.

## Data availability

All data are contained within this article and in the supporting information.

## Supporting information

This article contains supporting information.

## Conflicts of interest

The authors declare that they have no conflict of interest with the contents of this article.
