# Supplementary material for: Pentacyclic triterpenoid ursolic acid interferes with mast cell activation via a lipid-centric mechanism affecting FcεRI signalosome functions
Source: J Biol Chem. 2022 Sep 15;298(11):102497. doi: 10.1016/j.jbc.2022.102497 (PMC9587013; doi:10.1016/j.jbc.2022.102497)
Supplement: Supporting information [file mmc1.docx]

**Supporting information**

**Pentacyclic triterpenoid ursolic acid interferes with mast cell activation by a lipid-centric mechanism affecting FcεRI signalosome functions**

**Gouse M. Shaik, Lubica Draberova, Sara Cernohouzova, Magda Tumova, Viktor Bugajev, and Petr Draber**

Corresponding author: Petr Draber; e-mail: [draberpe@img.cas.cz](mailto:draberpe@img.cas.cz)

The supporting information contains supplemental figures which show the effect of UA on antigen-induced tyrosine phosphorylation of:

Lyn kinase (Fig. S1).

proteins in detergent-resistant membrane microdomains (Fig. S2).

**
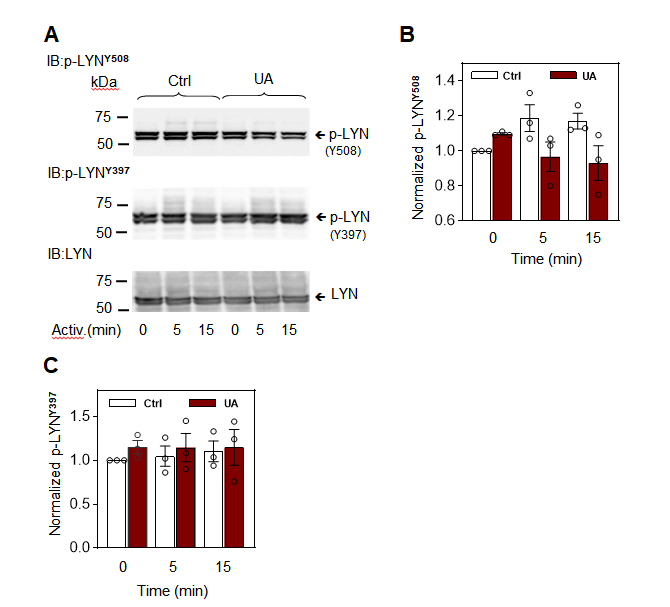
**

**Fig. S1. Pretreatment with UA has no effect on tyrosine phosphorylation of LYN kinase.** IgE-sensitized BMMCs were preincubated for 15 min with vehicle (0.1% DMSO; Ctrl) or 50 µM UA and then activated or not for the indicated time intervals with antigen (TNP-BSA; 250 ng/ml). (A) Whole-cell lysates were analyzed by immunoblotting for tyrosine phosphorylation of LYN (p-LYN^Y508^ or p-LYN^Y397^) and LYN, used as a loading control. Representative immunoblots from three independent experiments are shown. The results in (**B** and **C**) show densitometry analyses of the corresponding immunoblots in which signals from tyrosine-phosphorylated proteins in activated cells are normalized to the signals in nonactivated cells and loading control proteins. Means ± SEMs were calculated from three independent experiments.


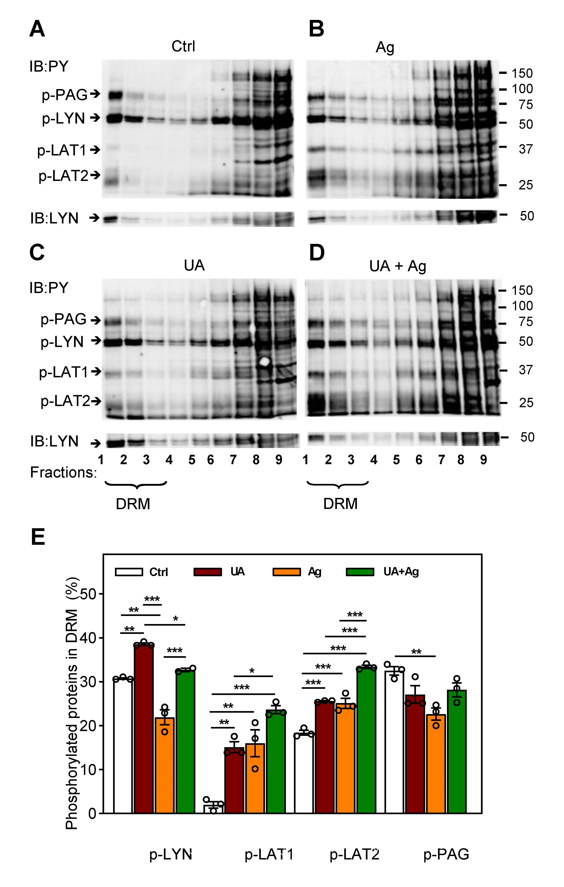


**Fig. S2. UA-induced changes in the distribution of tyrosine-phosphorylated proteins in the plasma membrane microdomains.** (**A** - **D**) IgE-sensitized BMMCs were pretreated for 15 min with vehicle (0.1% DMSO; **A** and **C**) or with 50 µM UA (**B** and **D**) and nonactivated (**A** and **B**) or activated (**C** and **D**) with antigen (Ag; TNP-BSA; 250 ng/ml). After 5 min activation, the cells were solubilized in 1% Brij-96-containing lysis buffer and then fractionated in a sucrose density gradient. Individual fractions were collected from the top of the gradient, starting from fraction 1. Proteins in the fractions were size-fractionated by SDS-PAGE and examined for tyrosine phosphorylation by immunoblotting with the PY-20-HRP conjugate. Arrows indicate positions of phosphorylated PAG, LYN, LAT1, and LAT2. The distribution of LYN kinase in individual fractions is shown at the bottom. Fractions 1-3, containing DRMs, are also marked. The numbers on the right indicate the positions of molecular weight markers in kDa. Representative immunoblots from each group are shown. (E) All immunoblots were analyzed by densitometry, and the relative amounts of phosphorylated LAT1, LAT2, LYN, and PAG in DRMs were determined. Means ± SEMs were calculated from three independent experiments. Statistical significance of intergroup differences is also shown.
